# Oropharyngeal detection of specific gut-derived Enterobacterales is associated with increased respiratory infection risk in older adults

**DOI:** 10.3389/fragi.2025.1566034

**Published:** 2025-05-30

**Authors:** Sophie J. Miller, Frank Zhang, Steven L. Taylor, Andrew P. Shoubridge, Erin Flynn, Egi Vasil, Richard J. Woodman, Lito E. Papanicolas, Geraint B. Rogers

**Affiliations:** ^1^ Microbiome and Host Health Program, South Australian Health and Medical Research Institute, Adelaide, SA, Australia; ^2^ College of Medicine and Public Health, Flinders University, Bedford Park, SA, Australia; ^3^ Microbiology and Infectious Diseases, SA Pathology, Adelaide, SA, Australia; ^4^ Flinders Health and Medical Research Institute, College of Medicine and Public Health, Flinders University, Adelaide, SA, Australia

**Keywords:** older people, nursing homes, pneumonia, microbiome, Enterobacterales

## Abstract

Respiratory tract infections (RTI) are a major contributor to morbidity and mortality in later life. RTI risk factors in older populations, including declining general health, altered airway physiology, and increased pharmaceutical exposures, also contribute to changes in the oropharyngeal (OP) microbiota. We sought to investigate whether such changes predict future incidence of RTI. OP microbiota characteristics were measured in 190 residents of long-term aged care. Fifty-four participants (28.4%) experienced one or more study-defined RTIs during the 12-month follow-up period, of which 28 (14.7%) occurred within 90 days of sample collection. OP microbiota composition was significantly associated with days to RTI event (F = 1.74, R^2^ = 1.02%, p = 0.04). Detection of Enterobacterales species (*Enterobacter cloacae*, *Escherichia coli*, *Klebsiella oxytoca*, *Klebsiella pneumoniae*, *Klebsiella variicola*, and *Proteus mirabilis*) were independently associated with RTI risk after covariate adjustment (subdistribution HR: 4.84; 95% CI: 1.65–14.19; p = 0.002). Strain-level analysis performed on metagenomes from contemporaneous OP and stool samples identified co-carriage of indistinguishable Enterobacterales strains in those with Enterobacterales-positive OP samples, suggesting intra-participant strain acquisition. We report OP carriage of Enterobacterales species to be a marker of future RTI risk in long-term aged care residents, reflecting the independent influence of common ageing-associated risk exposures.

## Introduction

The risk of serious complications arising from respiratory tract infection (RTI), including hospitalisation and death, increase considerably after the age of 65 years (Kochanek et al., 2019). The vulnerability of older individuals to RTI reflects a range of factors, including changes in airway physiology (Chong and Street, 2008) and immune function (Chong and Street, 2008; Simmons et al., 2021) that arise as a consequence of both biological ageing and external exposures, such as polypharmacy (Torres et al., 2017; Ho et al., 2001). These factors contribute to an increased likelihood of both RTI development and poor clinical outcomes.

Consideration of upper airway bacterial communities is important if we are to better understand RTI risk in older populations. Extrinsic factors, such as pharmaceutical exposures, and intrinsic factors, such as ageing-associated changes in airway physiology, can both influence oropharyngeal (OP) microbiota characteristics (DeClercq et al., 2021; Shigdel et al., 2023; Lee et al., 2019). Beyond providing a marker of risk exposures, such changes might also contribute to the development of RTI directly. Upper respiratory microbiota are important reservoirs for pathobiont species, including *Streptococcus pneumoniae*, *Haemophilus influenzae*, and *Staphylococcus aureus*, Group A *Streptococcus*, *Klebsiella pneumoniae* and *Moraxella catarrhalis* (Torres et al., 2014; Man et al., 2017). Disruption of the upper respiratory commensal microbiota, as a consequence of viral infection or antimicrobial treatments, for example, can result in pathobiont proliferation, increased local inflammation, and a greater risk of pathobiont translocation into the lower airways (Man et al., 2017; Lapidot et al., 2022; Pulvirenti et al., 2024).

We therefore hypothesised that OP microbiota characteristics are associated with relative RTI risk in older adults. To test this, we characterised the OP microbiota of participants of the *Generating evidence on Resistant bacteria in the Aged Care Environment* (GRACE) study; a cross-sectional study of microbial determinants of health in residents of long-term aged care facilities (Carpenter et al., 2023). The relationship between OP microbiota traits, RTI risk exposures, and RTI incidence during the 12-month follow-up was explored.

## Methods

### Study design, participant recruitment and data collection

GRACE was an observational cohort study involving five long-term aged care facilities in metropolitan South Australia, conducted between March 2019 and March 2020 (Carpenter et al., 2024). All study site residents were assessed for eligibility. Those in respite care (non-permanent care), end-of-life care, or without a contactable next of kin, were excluded. Other potential participants were excluded at the managers discretion. Participants provided OP swabs and stool samples, and consented to data relating to medication use, healthcare service utilisation (including pathology services), and comorbidities. These data were sourced from facility records, the Pharmaceutical Benefits Scheme (PBS), Medicare Benefits Schedule (MBS), Aged Care Funding Instrument (ACFI) assessments (Australian Government, 2016) and the Rx-Risk comorbidity tool (Pratt et al., 2018).

### Data definitions

The assessed clinical outcome was 12-month RTI incidence following sample collection. RTI was defined according to three factors: 1) radiographically proven pneumonia, as documented in participants’ electronic medical record (eMR) during hospital presentation; 2) a clinical diagnosis of respiratory tract infection of any type, made at hospital in the absence of clear radiographic evidence, also recorded in the eMR; or 3) dispensation of a short-course respiratory antibiotic (amoxicillin, doxycycline, erythromycin, azithromycin, clarithromycin, or roxithromycin) as recorded by the PBS. An RTI was considered to have occurred if an individual met one or more of these criteria.

The study period (April 2019 to February 2021) coincided with the early stages of the COVID-19 pandemic. However, no confirmed cases of COVID-19 were reported among aged care residents in South Australia prior to the completion of the study (Australian Government, 2024a).

Mortality data was derived from the death registry. Comorbidities were determined using ACFI assessment data and the Rx-Risk Comorbidity Index based on PBS prescription data (Sloan et al., 2003). Pre-existing lung disease (asthma, chronic obstructive pulmonary disease, obstructive sleep apnoea, bronchiectasis, chronic endobronchial obstruction with lung collapse, or lung cancer) was indicated by either eMR or inhaler prescription based on PBS.

### Sample collection and metagenomic analysis

Detailed information about sample processing is described in the Supplementary Material. Briefly, OP samples were collected using FLOQSwabs (Copan, CA, United States) pressed over the tonsils and posterior pharyngeal wall, avoiding teeth and gingiva on withdrawal. Swabs were placed in 2 mL tubes containing 400 µL of Tris-EDTA (TE) buffer (Invitrogen, CA, United States) and immediately stored at −80°C. DNA from swabs was extracted using the ZymoBIOMICS DNA Miniprep Kit (Zymo Research, CA, United States), which combines physical lysis with bead beating and chemical lysis, followed by column-based purification to ensure high-quality DNA recovery. Stool samples were collected into Norgen Nucleic Acid Collection and Preservation Tubes (Norgen, ON, Canada) and DNA extracted using the PowerLyzer PowerSoil DNA Isolation Kit (Qiagen, Hilden, Germany), as previously described (Miller et al., 2024). Both OP and stool samples underwent metagenomic library preparation using the Nextera XT DNA Library Prep Kit (Illumina, CA, United States) as per manufacturer’s instructions, alongside blank and mock community controls. Libraries were normalised with Qubit assay, and pooled libraries were then sequenced on an Illumina Novaseq 6,000 platform with a 2 × 150 setup. Metagenomic sequence data are accessible via the European Nucleotide Archive (ENA) under the accession number PRJEB51408.

Sequences were quality-filtered using Trimmomatic (v0.39) and reads that aligned to the NCBI human reference genome (release GRCh38) were removed using Bowtie (v2.3.5.1) (Bolger et al., 2014; Langmead and Salzberg, 2012). The resulting quality filtered, non-human reads had a median count of 47.8 million read pairs per OP sample (range: 33.6–56.7) and 44.4 million read pairs (range: 33.2–54.7) per stool sample. Microbiome taxonomic composition data was determined using MetaPhlAn (v3.0) (Beghini et al., 2021). Alpha diversity metrics (species richness, Shannon’s diversity, and Pielou’s evenness index) were calculated from taxonomic relative abundances at the species level using the vegan R package (Oksanen et al., 2022). The distance from centroid (the distance between a sample and the corresponding group centroid) was calculated from Bray-Curtis dissimilarity. The oxygen tolerance of species was determined using Bac*Dive* (December 2022 release) (Reimer et al., 2021). The presence and relative abundance of strains belonging to *Escherichia coli*, *K. pneumoniae*, *Klebsiella variicola* and *Klebsiella oxytoca* in paired OP swabs and stool samples were determined using the StrainGST module of Strain Genome Explorer (StrainGE, v1.3.9) (van Dijk et al., 2022). Reference databases were generated from RefSeq complete genomes, with clustering performed at a default threshold of 0.9 Jaccard similarity, which corresponds to an approximate average nucleotide identity (ANI) of 99.8%.

### Statistical analysis

All statistical analyses accounted for potential confounding factors influencing OP microbiome characteristics and RTI incidence. Covariate selection for multivariable modelling was informed by univariate assessments of age, sex, days since entry to nursing home, RTI in preceding 12 months (defined by PBS prescription of a short-course respiratory antibiotic), any antibiotic dispensation within the 90 days before sample collection, proton-pump inhibitor (PPI) dispensation within the prior 90 days, inhaled corticosteroid (ICS) dispensation within the prior 90 days, lung disease, the number of recorded Rx-Risk comorbidities, and dietary modification status based on the International Dysphagia Diet Standardisation initiative (IDDSI) framework (Cichero et al., 2017) (Supplementary Table S1). The IDDSI framework was used as a proxy for swallowing function and dietary modification needs, as more detailed nutritional intake data were not available.

Inter-group differences in microbiota composition (beta diversity) were determined by Permutational Multivariate Analysis of Variance (PERMANOVA) on square-root transformed Bray-Curtis dissimilarity distances, with 9,999 random permutations, using the function ‘adonis2’ from the vegan R package (v2.6–4) (Oksanen et al., 2022). Differences in multivariate dispersion of microbiome composition between groups were assessed using the ‘betadisper’ function from the vegan package. To adjust for the covariates previously described, residuals were extracted from a multivariate linear model fitted to the square-root-transformed abundance data. These residuals were shifted to ensure non-negativity and used to construct a Bray-Curtis distance matrix. Statistical significance was tested with the ‘permutest’ function (9,999 permutations), and results were reported as the F-statistic and p-value.

Alpha diversity and distance-to-centroid metrics were ranked into quartiles for analysis. Taxa at the order level were analysed using ranked quartiles when detection was ≥30%, and presence/absence data when detection was <30%. Screening of order-level taxa for association with RTI risk was conducted using a univariate Fine-Gray subdistribution hazard model, with a two-sided Type I error rate (α = 0.05), accounting for death as a competing event (Fine and Gray, 1999). Associations between OP microbiota characteristics and RTI risk were assessed using a multivariable Fine-Gray subdistribution hazard model, adjusting for covariates previously described. Subdistribution hazard ratios (SHR) and 95% confidence intervals (CI) are reported.

Principal Coordinates Analysis (PCoA) plots were generated using Bray-Curtis dissimilarity of square-root transformed relative abundance data, using vegan (Oksanen et al., 2022). Phylogenetic trees were constructed using snippy (v4.6.0) and FastTree (v2.1.11), and visualised using FigTree (1.4.4) (Price et al., 2010; Seemann, 2015; Rambaut, 2018).

## Results

### Cohort characteristics

OP swabs and healthcare utilisation data were available for 190 long-term aged care residents. Participant median age was 88.3 years (IQR 81.3–92.6 years) and 71.1% of the cohort were female. The median period of aged care residency prior to sample collection was 597.5 days (IQR 259–1,031 days). Thirty-five participants died during 12-month follow-up period (18.4%), of whom eleven (5.8%) died within the 90 days following sample collection.

Fifty-four participants (28.4%) received a study-defined RTI diagnosis during the 12-month follow-up, with 28 (14.7%) falling within 90 days of sample collection. Of those participants who received an RTI diagnosis in the 12-month follow-up period, 40 (74.1%) experienced one RTI, six (11.1%) experienced two RTIs, five (9.3%) experienced three RTIs, two (3.7%) experienced four RTIs, and one (1.9%) experienced seven RTIs.

The characteristics of participants who experienced an RTI during the 12-month follow-up differed from those who did not (Table 1). Diagnosis was associated with a higher incidence of pre-existing lung disease (55.6% vs. 28.7%), dispensation of short-course respiratory antibiotics (50% vs. 24.3%), dispensation of any antibiotic (51.9% vs. 31.6%), ICS (18.5% vs. 5.9%) and PPI use during the prior 90 days (59.3% vs. 40.4%). Those diagnosed with one or more RTI also exhibited a greater burden of comorbidities, as evidenced by higher Rx-Risk scores (median 7, IQR 5–8), compared to no RTI (median 4, IQR 2–7).

**TABLE 1:** Cohort characteristics.

Variable	12-month RTI	No RTI
N (% total cohort)	54 (28.4)	136 (71.6)
Age (years), median [IQR]	88.8 [81.2–92.8]	88.3 [81.8–92.6]
Female sex, n (%)	38 (70.4)	97 (71.3)
Duration of residence, median (IQR)	543 [301–861]	653 [242–1,082]
All-cause mortality, n (%)		
90-day	3 (5.6)	8 (5.9)
12-month	13 (24.1)	22 (16.2)
Comorbidities		
Lung disease, n (%)	30 (55.6)	39 (28.7)
Prior respiratory infection (12 months), n (%)	27 (50)	33 (24.3)
Modified diet, n (%)	9 (16.7)	33 (24.3)
Number of Rx-Risk comorbidities, median [IQR]	7 [5–8]	4 [2–7]
Medications dispensed within 90 days prior to sample collection, n (%)		
Antibiotics	28 (51.9)	43 (31.6)
Inhaled corticosteroids	10 (18.5)	8 (5.9)
Proton pump inhibitors	32 (59.3)	55 (40.4)
Antipsychotics	8 (14.8)	13 (9.6)
Sedatives	25 (46.3)	51 (37.5)
Antihypertensives	37 (68.5)	81 (59.6)
Antiparkinsonians	8 (14.8)	16 (11.8)

### OP microbiota characteristics of aged care residents and association with RTI

Thirty-one bacterial orders consisting of 322 distinct species were identified within the cohort OP metagenome (Supplementary Figure S1A), most commonly including species often found in the oropharynx, such as *Actinomyces*, *Gemella*, *Prevotella*, *Rothia*, *Streptococcus*, and *Veillonella spp* (Supplementary Figure S1B). A total of 131 detected species were classed as anaerobic.

**FIGURE 1:**
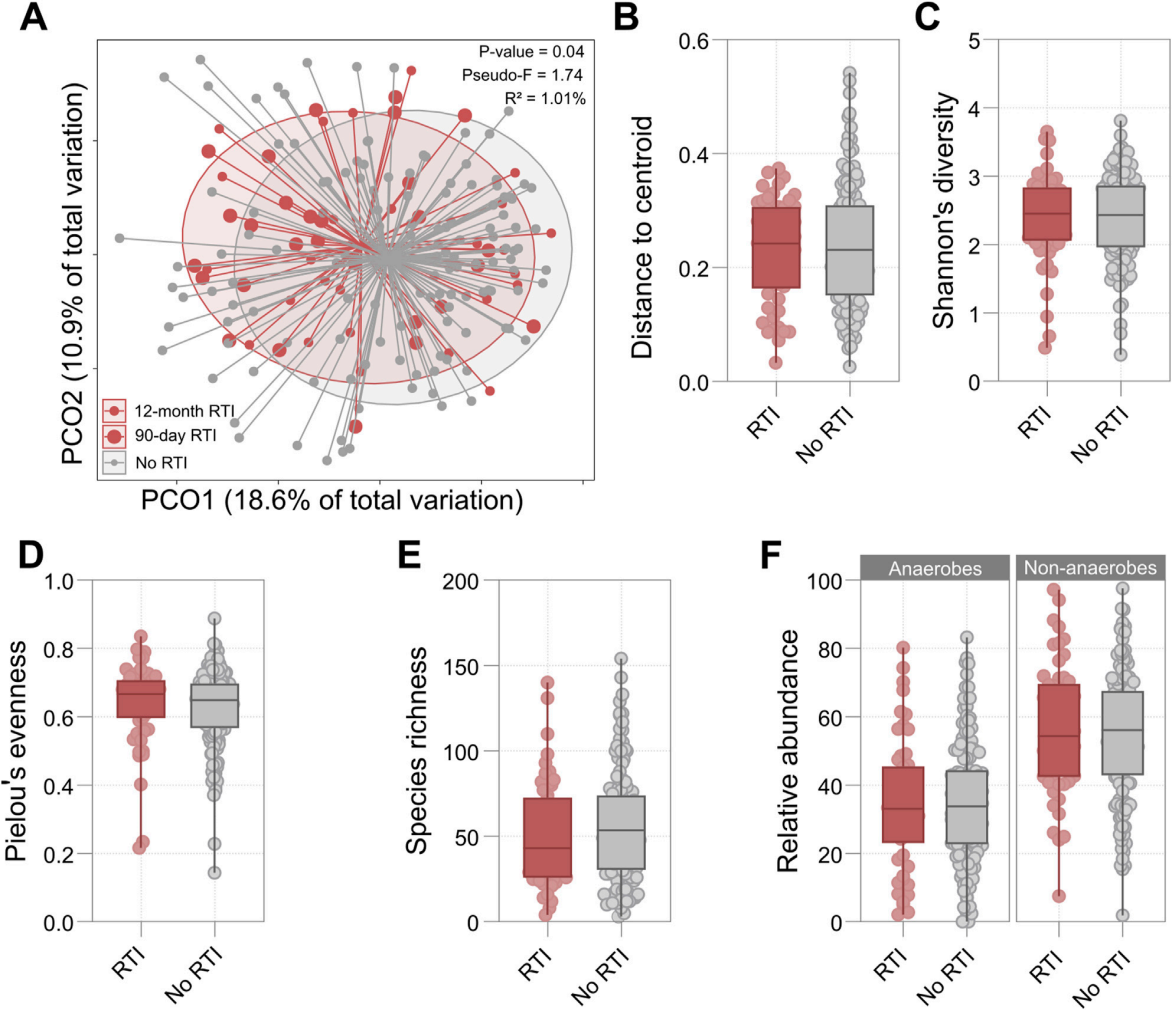
**(A)** Principal Coordinates Analysis (PCoA) plot based on Bray-Curtis distances comparing participants who developed RTI in the 12-month follow-up period and those who did not. Large red points represent RTI cases at 90 days post-collection (n = 28), small red points indicate RTI cases at 12 months post-collection (n = 54), and grey points show participants who did not develop RTI (n = 136). The p-value, pseudo-F statistic, and PERMANOVA-derived explained variance (R^2^) for the association between 12-month RTI status and beta diversity are presented. **(B–F)** Box plots comparing microbial diversity metrics between participants who developed RTI in the 12 months following sample collection (red) and those who did not (grey): **(B)** Distance to centroid, **(C)** Shannon’s diversity index, **(D)** Pielou’s evenness, **(E)** Species richness, **(F)** Relative abundance of anaerobes and non-anaerobes in RTI and non-RTI groups. For all box plots, the central line represents the median, boxes show interquartile ranges, and whiskers extend to the range. Details of statistical output are provided in Supplementary Table S3.

OP microbiota beta diversity was significantly associated with future RTI. While this relationship was strongest when analysis was restricted to RTIs during the 90 days following sample collection (F = 2.23, R^2^ = 1.29%, p = 0.01), it remained significant when analysis was extended to the full 12-month follow-up period (F = 1.74, R^2^ = 1.01%, p = 0.04) (Figure 1A). The association between microbiota composition and RTI was also significant when time to RTI was treated as a continuous variable (F = 1.76, R^2^ = 1.02%, p = 0.04). Bray-Curtis distance from centroid and permutational dispersion analysis was not associated with RTI diagnosis during the 12-month follow-up period (Figure 1B; Supplementary Table S2), nor were alpha diversity measures (species richness, Shannon’s diversity, and Pielou’s evenness) (Figures 1C–E; Supplementary Table S2). Furthermore, neither the ranked cumulative abundance of anaerobic species nor the ranked cumulative abundance of non-anaerobic species was associated with the incidence of RTI during the 12-month follow-up period (Figure 1F; Supplementary Table S2). No significant associations were observed when analyses were restricted to RTIs during the 90 days following sample collection (Supplementary Table S2).

### Detection of Enterobacterales in OP microbiota was associated with future RTI

**FIGURE 2:**
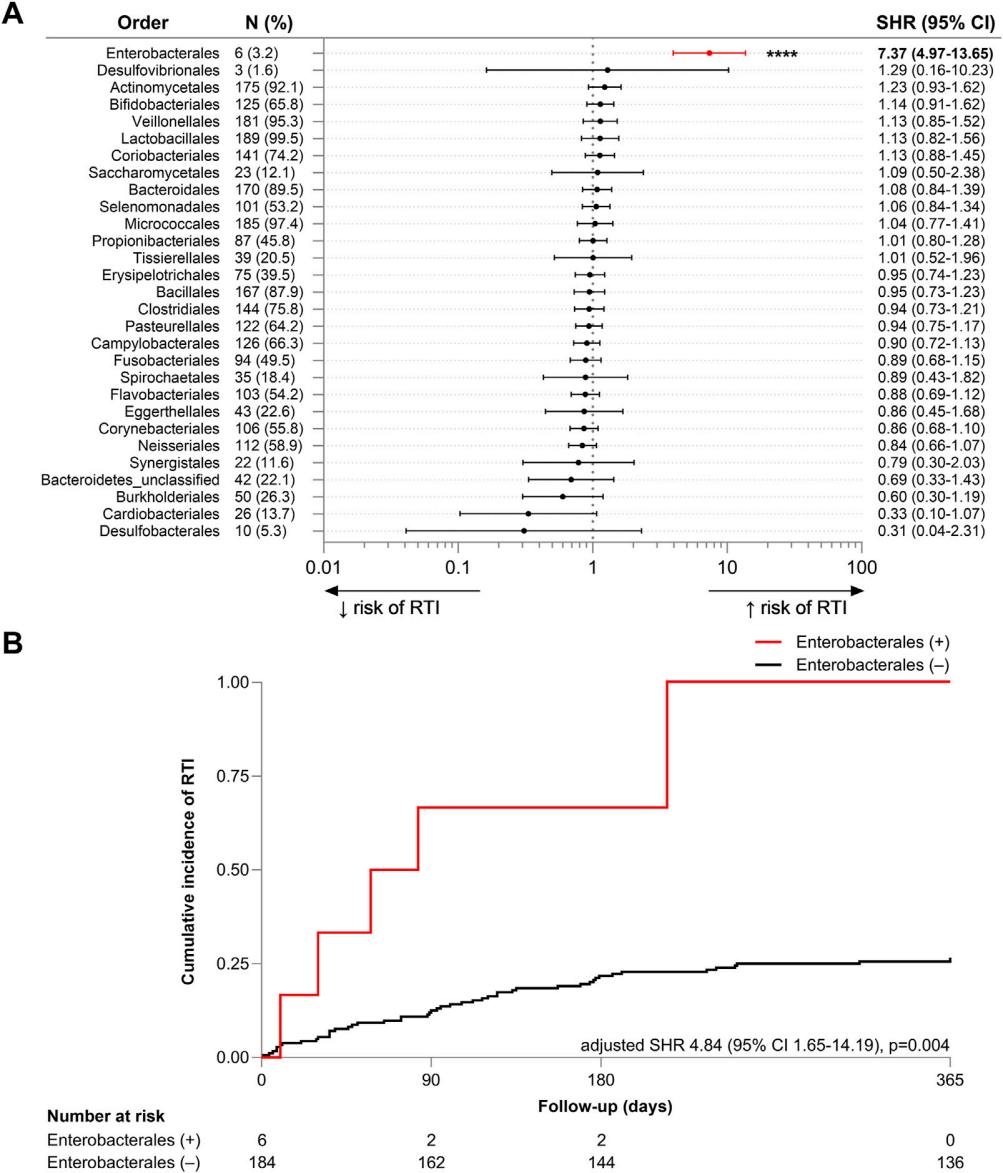
**(A)** Forest plot of univariate subdistribution hazard ratios (SHRs) for the association between the OP detection of bacterial orders and risk of RTI in the 12-month follow-up period. The plot includes the SHR with 95% confidence intervals (CI) for each bacterial order, with n (%) indicating the number and proportion of participants in whom each order was detected. **(B)** Cumulative incidence plot of RTI incidence over the 12-month follow-up period (days) in participants with (red, +) and without (black, −) detection of bacterial members of the Enterobacterales order. Fine-Gray subdistribution hazard model was adjusted for mortality, age, sex, duration of residence, number of comorbidities, existing diagnosis of lung disease, modified diet, respiratory antibiotic use within 12 months prior to sample collection, and dispensation of any antibiotic, inhaled corticosteroid, or proton pump inhibitor within the prior 90 days. Adjusted subdistribution hazard ratio (SHR), 95% confidence interval (CI), log-rank chi-square and p-value is reported. The number of participants at risk of RTI, classified by status of Enterobacterales detection, is reported. ****p < 0.0001.

To investigate whether taxonomic differences were associated with future RTI, univariate analysis was performed on the 30 bacterial orders detected. A significant positive association was found between the detection of Enterobacterales in the oropharynx and the diagnosis of future RTI (SHR 7.37, 95% CI 3.97–13.65, p < 0.001) (Figure 2A). Specific species within this order, including *Enterobacter cloacae*, *E. coli*, *K. oxytoca*, *K. pneumoniae*, *K. variicola*, and *Proteus mirabilis*, were detected in six participants (3.2%). After adjusting for covariates, the association remained significant (adjusted SHR 5.12, 95% CI 1.84–14.25, p = 0.002) (Figure 2B).

The six individuals who were OP Enterobacterales-positive all developed one or more RTI during the 12-month follow-up period (four within 90 days). Four of these six individuals were male, four had pre-existing lung disease (66.7%, compared to 54.2% within the wider RTI cohort), and four had experienced RTI in previous 12 months (66.7% vs. 47.9%). Two had a dispensing history that included ICS (33.3% vs. 16.7%), four that included PPIs (66.7% vs. 58.3%), and three that included an antibiotic (50% vs. 52.1%) during the prior 90 days. The co-occurrence of these characteristics is presented in Supplementary Table S3 and Supplementary Figure S2.

### OP Enterobacterales strains were detectable in paired faecal samples

**FIGURE 3:**
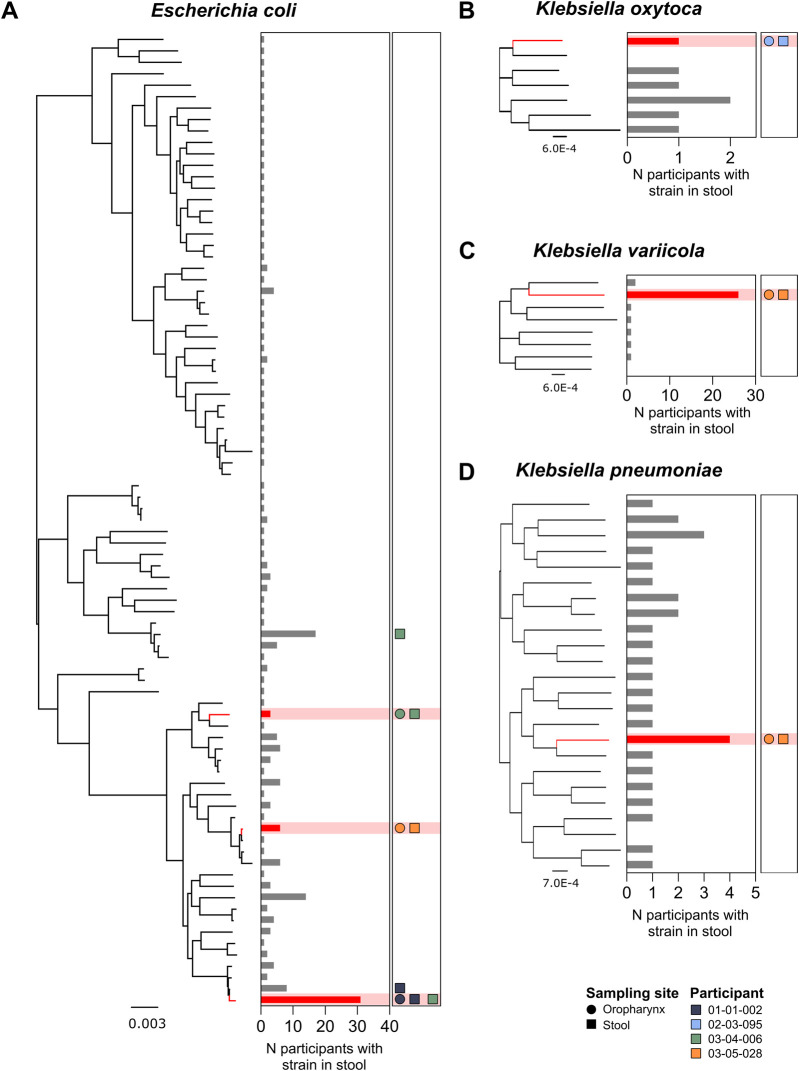
Phylogenetic distribution of *E. coli* **(A)**, *K. oxytoca* **(B)**, *K. variicola* **(C)**, and *K. pneumoniae* **(D)** strains identified in all stool samples from participants with a paired OP swab (n = 139 participants). Red nodes represent Enterobacterales strains detected in paired OP swabs and stool samples. Bar plots illustrate the total number of participants with the corresponding strain present in stool samples. Shapes indicate the detection of strains in the four participants with Enterobacterales species present, where each colour represents one participant. Circles indicate the detection of the corresponding strain in the oropharynx, while squares represent detection in stool.

In addition to an oropharyngeal swab, 139 study participants provided a stool sample. This subgroup included four of the six individuals who were OP Enterobacterales-positive. Six discrete Enterobacterales strains were detected in the OP swabs of these four participants; 3 *E. coli* strains [Nissle 1917 (GCA_021559835.1), SCU-124 (GCA_012934635.1), M1/5 (GCA_013390265.1)], 1 *K. oxytoca* strain [86 (GCA_033864495.1)], 1 *K. variicola* strain [FF907 (GCA_023703815.1)], and 1 *K. pneumoniae* strain [INF125 (GCA_003432285.1)] (Figures 3A–D, respectively). In each case, an undifferentiable strain (ANI ≥99.8%) was detected in the corresponding stool sample from the same individual (Supplementary Table S4).

In four of these six cases, the Enterobacterales strain identified in the oropharynx was the only strain belonging to that species that was detectable within the matching stool sample; these included one individual in whom *E. coli*, *K. variicola,* and *K. oxytoca* were detected in their OP swab. In two cases, both involving *E. coli*, additional strains were also detectable in the corresponding stool sample (Figure 3A).

## Discussion

We report a significant relationship between OP microbiota characteristics and prospective RTI incidence in residents of long-term aged care. Specifically, we identify an association between RTI diagnosis and both altered beta diversity and carriage of specific Enterobacterales species in the oropharynx.

The observed association between OP microbiota diversity and RTI risk is consistent with previous research relating to upper respiratory microbiota traits and infectious disease outcomes in older individuals. For example, de Steenhuijsen Piters and colleagues reported significant differences in OP microbiota composition in older individuals with pneumonia compared to an age-matched healthy control group. The authors identified shifts in overall microbial community structure, alongside notable reductions in species richness and an increased prevalence of certain pathobiont taxa, such as *S. pneumoniae* (de Steenhuijsen Piters et al., 2016). Consistent, but less pronounced, differences were also identified between a younger adult cohort with pneumonia and age-matched healthy controls. In both contexts, pneumonia was associated with a depletion of certain anaerobic taxa common in the oropharynx and oral cavity; a finding that could be attributed, at least in part, to chronic oxygen therapy and exposure to antibiotics with anti-anaerobic activity (Chanderraj et al., 2023; Kitsios et al., 2023). However, it is notable that the authors identified differences in microbiota composition not only between those with pneumonia and healthy controls, but also between young and older healthy cohorts, suggesting a potential direct contribution of ageing-associated changes in OP microbiota to increased risk of lower respiratory infection in older adults (de Steenhuijsen Piters et al., 2016).

A population-based analysis undertaken by the same group identified a gradual decline in the microbial diversity of the oropharyngeal microbiota during adulthood, with a significantly lower diversity evident in older participants (65 years and above) compared with adults (Odendaal et al., 2024). Other investigators have reported similar patterns of OP microbiota change in later life (Liu et al., 2024; Whelan et al., 2014), as well as links between poorer respiratory health and the carriage of certain pathobiont genera, such as *Moraxella*, in older individuals (van den Munckhof et al., 2020).

Such changes in wider upper airway microbiota characteristics appear to be broadly conserved within older populations, and are consistent with our observation of a significant association between OP microbiota beta diversity and subsequent RTI within the study cohort. However, no association between microbiota dispersion or decreased alpha diversity and RTI risk was identified within our study population. A number of factors potentially contribute to the observed changes in OP microbiota structure in older populations. Common exposures in later life, particularly increasing polypharmacy, may act to disrupt or deplete commensal microbial communities in the upper airways. Such perturbation can contribute to a reduction in microbiota resistance and resilience; broadly defined as the extent to which microbial composition changes in response to a disturbance and the degree to which baseline characteristics are re-established afterwards, respectively (Cuthbertson et al., 2016). Additionally, such disruption appears to contribute to reduced differences in microbiota composition between the oropharynx and the anterior nares, as compared to mid-aged adults (Whelan et al., 2014).

In addition to extrinsic exposures, biological ageing also contributes to OP microbiota change. Investigations utilising preclinical models suggest that patterns of mucin production and glycosylation, which represent important contributors to microbial energy and carbon sources in the airway, also change with age (Tian et al., 2023). Such changes in airway physiology, including local immune homeostasis, are likely to act as a feedback loop for microbiota regulation. Commensal microbiota in the airways are known to regulate, for example, the generation of virus-specific CD4^+^ and CD8^+^ T cells and antibody responses following respiratory influenza virus infection, a process that is disrupted by various antibiotic exposures (Ichinohe et al., 2011).

In our study population, OP microbiota characteristics linked to increased RTI risk did not include an increased prevalence of pathobiont species commonly associated with acute respiratory infections, such as *S. pneumoniae* and *H. influenzae*. Instead, we observed a significant association between RTI risk and oropharyngeal carriage of specific enteric bacteria of the Enterobacterales order, including *E. coli*, *E. cloacae*, *P. mirabilis*, and *Klebsiella* species. This finding is particularly notable given the high rates of pneumonia caused by these bacteria in older adults, especially those who are frail or living with cognitive impairment (El-Solh et al., 2003; El-Solh et al., 2004; Janssens and Krause, 2004).

There are a number of potential explanations for this finding. Although direct evidence for increased faecal-oral transmission of enteric organisms in later life remains limited, individuals with greater personal care needs, such as those in long-term care facilities requiring assistance with both oral and personal hygiene tasks like dressing, washing, and grooming, face a heightened risk of healthcare-associated infections, including those involving enteric bacteria (El-Solh et al., 2004; Australian Government, 2024b). In these settings, challenges in maintaining consistent hygiene practices may contribute to the colonisation of the upper respiratory tract by enteric organisms. The detection of identical Enterobacterales strains in OP swabs and contemporaneous stool samples further supports the hypothesis that faecal-oral transmission may play a role in the upper respiratory tract colonisation observed in older long-term care residents. In addition to faecal-oral transmission, the widespread use of medications that suppress gastric acid, particularly proton pump inhibitors, could also contribute to increased rates of aspiration and OP carriage of enteric microbes (Rosen et al., 2014; Meijvis et al., 2011; Fohl and Regal, 2011).

While our findings do not establish a causal relationship between OP Enterobacterales carriage and RTI development, it is possible to speculate about what this phenomenon is likely to represent. As described above, multiple factors contribute to both changes in OP microbiota composition and increased RTI risk, including ageing and polypharmacy-associated airway pathophysiology, loss of microbiota integrity, increased faecal-oral transmission, and altered immune function, independently. At the same time, the likelihood that the taxa we identified as markers of RTI risk contributed directly to RTI development is low according to our current understanding of their pathogenic potential and existing pathology data relating to RTI causation in older adults. Instead, we interpret the presence of OP Enterobacterales as an indicator of broader host-related vulnerabilities that predispose individuals to respiratory infections, rather than as a direct causal factor. This interpretation aligns with existing evidence suggesting that microbiota alterations in older adults frequently reflect underlying frailty, immune dysregulation, and cumulative health burdens, all of which independently increase RTI susceptibility (Whelan et al., 2014; Claesson et al., 2012). As such, a conservative interpretation would be that direct and indirect consequences of biological ageing result in the alteration of OP microbiota in a manner that both promotes Enterobacterales carriage and, independently, a greater susceptibility to poor respiratory health outcomes. Consequently, longitudinal or interventional studies would be required to determine whether modifying Enterobacterales colonisation alters RTI risk.

Given these complexities, it may be more appropriate to consider OP Enterobacterales carriage within a broader framework of frailty and infection risk stratification, rather than as a direct target for intervention. Such an approach would prompt enhanced monitoring and preventative care. Specifically, in clinical practice, recognition of this increased vulnerability could prompt closer monitoring for early signs of RTI, assessment of swallowing function to reduce aspiration risk, optimisation of medication regimens, and reinforcement of oral and dental hygiene. By integrating microbiome-based risk assessment into broader frailty and infection prevention strategies, it may be possible to refine RTI risk prediction and improve outcomes for vulnerable aged care populations.

Our study had limitations that should be considered. First, the microbiota features that we report as associated with RTI in the cohort were relatively rare. As such, the presented findings require confirmation in a larger population. However, given that the need to recruit and obtain clinical samples and data access from individuals who are vulnerable and often experiencing significant cognitive decline, doing so will present challenges. Second, the approach to defining RTI cases that we employed (radiographic evidence of pneumonia, in-hospital diagnosis, or dispensation of a short-course respiratory antibiotic) is likely to miss instances of symptomatic RTI, particularly those of a less-severe nature or clear viral aetiology. Third, data relating to the causative agent in cases of RTI was not available, preventing an exploration of the potential direct involvement of OP pathobiont populations in respiratory infections. Fourth, the explanatory power of our beta diversity model was limited, which, while not uncommon in microbiome research, suggests that other important contributors to OP microbiota variation remain unmeasured. These may include nutritional status, smoking history, frailty severity, oral health, and other care-related exposures that were not available for inclusion in this study. Future studies that integrate richer clinical and behavioural metadata may enhance risk prediction and biological insight. Fifth, StrainGST relies on reference-based clustering using k-mer similarities, which may miss novel or divergent strains not in the reference database, potentially leading to misclassification or exclusion of distantly related strains. Additionally, strain detection was not performed on cultured organisms, which could limit the identification of certain strains or species.

In conclusion, carriage of Enterobacterales strains within the oropharynx appears to represent a marker of RTI risk in long-term aged care residents. While the basis for this association is not yet known, it may represent the parallel impacts of risk factors for microbiota alteration and RTI development that are common in later life.

## Data Availability

The datasets presented in this study can be found in online repositories. The names of the repository/repositories and accession number(s) can be found below: https://www.ebi.ac.uk/ena/browser/view/PRJEB51408; https://doi.org/10.6084/m9. figshare.27209472; https://github.com/sjmiller-sahmri/ GRACE-study.
