# Supplementary material for: Oropharyngeal detection of specific gut-derived Enterobacterales is associated with increased respiratory infection risk in older adults
Source: Front Aging. 2025 May 30;6:1566034. doi: 10.3389/fragi.2025.1566034 (PMC12162913; doi:10.3389/fragi.2025.1566034)
Supplement: Supplementary file 1 [file Table1.docx]

Supplementary Material

#

# Supplementary Methods

## Oropharyngeal swab DNA extraction

DNA from OP swabs was extracted using the ZymoBIOMICS miniprep kit (Zymo Research, CA, USA). Swabs were spun down at 3374g for 5 min to collect all biological material and the resultant solution was added to a bead-beating tube containing 750 μL of lysis buffer. Samples underwent bead-beating for 1 min 5 times at a speed of 6.5m/s in a FastPrep®-24 Homogenizer (MP Biomedicals, CA, USA) for a total of 5 min with 5 min rest in between each run. Samples were centrifuged at 10,000*g* for 2 min then 700 μL was added to the III-F filter in a clean tube and centrifuged at 8,000*g* for 1 min. Filtered solution was transferred to a clean tube and 2100 μL of DNA binding buffer was added. Samples were vortexed vigorously then 800 μL of solution was added to a IICR filter and centrifuged at 10,000*g* for 1 min. Flow through was discarded and this process was repeated until all solution had been passed through the filter. After transferring the filter to a new tube, 400 μL of the first wash buffer was added to the filter and centrifuged at 10,000*g* for 1 min. Flow through was discarded then 700 μL of a second wash buffer was added to the filter, centrifuged at 10,000*g* for 1 min and a final 200 μL of wash buffer was added to ensure all wash buffer had passed through. Filters were transferred to a new clean tube and 100 μL of dH2O at 60°C was added and incubated for 5 min. After centrifuging at 10,000*g* for 1 min, samples were added to a final spin column for purification and centrifuged at 16,000*g* for 3 min. DNA was stored at -80°C until further processing.

## Stool DNA extraction

DNA was extracted from stool samples using the Qiagen PowerLyzer PowerSoil DNA Isolation Kit (Qiagen, Hilden, Germany) as per the manufacturer’s instructions. Stool samples containing buffer were vortexed vigorously and 1 ml was transferred to a clean 2 mL tube. Samples were centrifuged for 20 min at 13,000*g* at 4°C and the supernatant was transferred to a clean 2 mL screw-cap tube for storage. The faecal pellet was combined with 750 μL of bead solution and transferred to a glass bead tube. After adding 60 μL of cell lysis buffer, samples were incubated at 65°C for 10 min. Samples underwent bead-beating in a FastPrep®-24 Homogenizer (MP Biomedicals, CA, USA) for 2 pulses of 1 min at 6.5m/s and were centrifuged at 10,000*g* for 3 min at room temperature. Supernatant was discarded and 250 μL of Inhibitor Removal Technology® (IRT) was added and vortexed for 5 s. Samples were incubated at 4°C for 10 min, centrifuged for 3 min at 10,000*g* at room temperature, and 600 μL of supernatant was transferred to a clean 2 mL tube. Precipitation reagent was combined with the supernatant and the sample was centrifuged for 3 min at 10,000*g* at room temperature again before transfer of 750 μL supernatant to another clean 2 mL tube. High concentration salt solution was added to the supernatant and vortexed for 5 s. Then 675 μL of supernatant was added to a Spin Filter and centrifuged for 10,000*g* for 1 min at room temperature. Flow through was discarded and this step was repeated 2 more times. 500 μL of ethanol-based wash solution was added to the spin column and centrifuged at 10,000*g* for 1 min at room temperature. Flow through was discarded and residual ethanol wash solution was removed from the spin column by a second centrifuge at 10,000*g* for 1 min. Spin columns were transferred to a clean 2 mL tube, 50 μL of UltraPure RNAse DNAse-free water was added and centrifuged for 1 min 10,000*g* at room temperature and repeated 2 more times to collect all DNA. Eluted DNA was stored at -80°C until further processing.

# Supplementary Figures and Tables

## Supplementary Figures

**
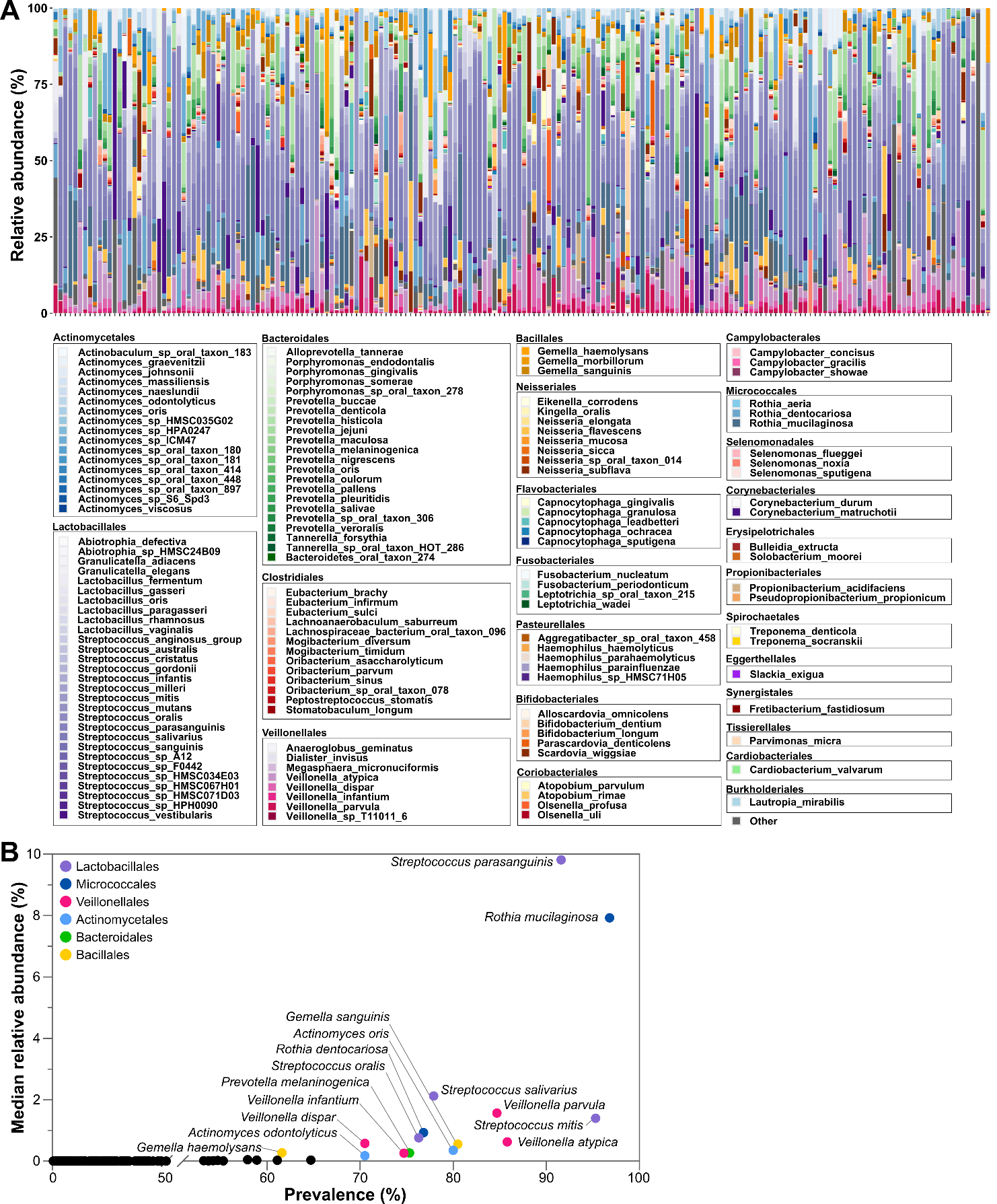
**

**Supplementary Figure 1.** **A** Taxa bar plot of all species detected in the cohort OP microbiota, by participant. Species are coloured by their phylogeny at the bacterial order level. The remaining less abundant species (detected in < 10% of participants) were grouped as a single bar (Other; grey).

**B** The frequency of bacterial species detected in the OP microbiome (in 190 participants) compared to their median relative abundances. Species are coloured according to order level.


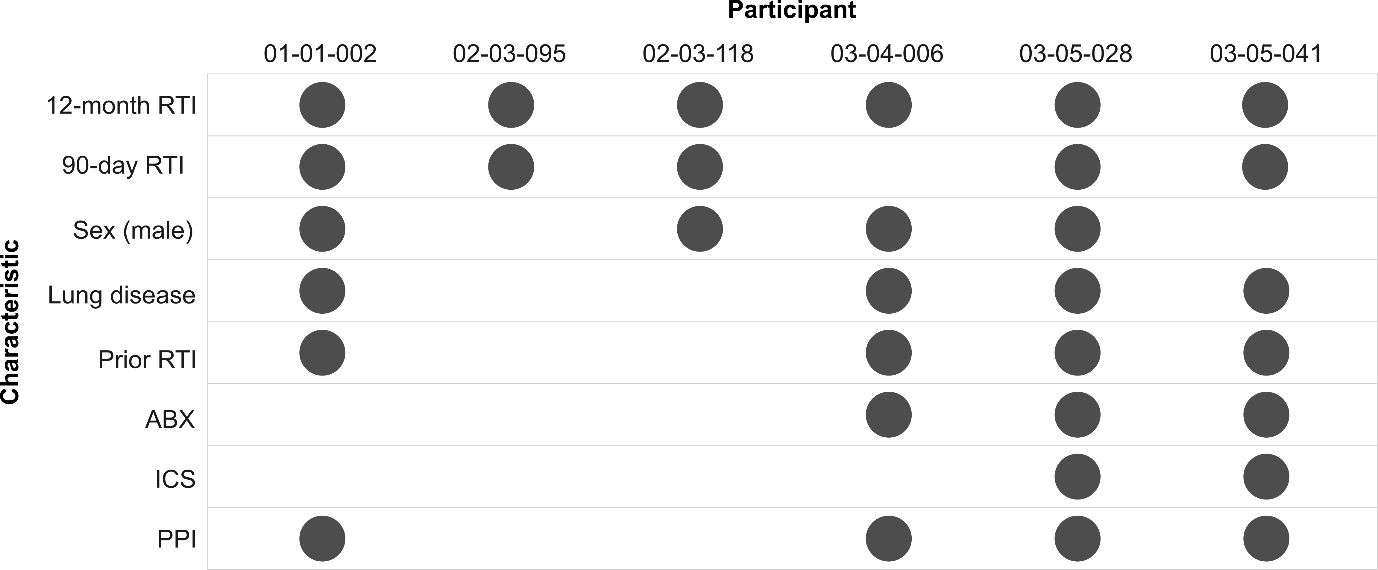


**Supplementary Figure 2.** Characteristics of participants (n=6) with Enterobacterales detected in their corresponding OP sample. Circles indicate the participant is positive for the associated characteristic, including RTI diagnosed within 90 days or 12 months from sample collection, male sex, lung disease, RTI diagnosed in the prior 12 months to sample collection, or prescription of antibiotics (ABX), inhaled corticosteroids (ICS), and proton pump inhibitors (PPI) in the previous 90 days to sample collection.

## Supplementary Tables

**Supplementary Table 1.** Cohort characteristics and risk of RTI within 12 months following sample collection. Univariate analysis was performed using Fine-Gray subdistribution hazard model with all characteristics. Hazard ratios (HR), corresponding 95% confidence intervals (CI), and respective p-values are presented.

| **Characteristic** | **HR (95% CI)** | **P value** |
| --- | --- | --- |
| Age | 1.01 (0.97-1.04) | 0.71 |
| Male sex | 1.07 (0.60-1.93) | 0.82 |
| Duration of residence | 1.00 (0.99-1.00) | 0.21 |
| **Comorbidities** |  |  |
| Lung disease | 2.46 (1.44-4.20) | **0.001** |
| Prior respiratory infection (12 months) | 2.40 (1.41-4.07) | **0.001** |
| Number of Rx-Risk comorbidities | 1.22 (1.12-1.34) | **<0.0001** |
| Modified diet | 0.67 (0.33-1.36) | 0.26 |
| **Medications dispensed within 90 days prior to sample collection** | | |
| Antibiotics | 2.00 (1.17-3.39) | **0.01** |
| Inhaled corticosteroids | 2.36 (1.27-4.38) | **0.01** |
| Proton pump inhibitors | 1.82 (1.05-3.14) | **0.03** |
| Antipsychotics | 1.69 (0.75-3.78) | 0.2 |
| Sedatives | 1.40 (0.82-2.39) | 0.21 |
| Antihypertensives (90 days) | 1.40 (0.79-2.48) | 0.25 |
| Antiparkinsonians (90 days) | 1.27 (0.60-2.71) | 0.53 |

**Supplementary Table 2**. Multivariate analysis examining the association between OP microbiome characteristics and RTI risk in both the 90-day and 12-month follow-up periods. Analyses were adjusted for age, sex, duration of residence, number of comorbidities, existing diagnosis of lung disease, modified diet, or prior respiratory infection within 12 months prior to sample collection; as well as dispensation of any antibiotic, inhaled corticosteroid, or proton pump inhibitor within the prior 90 days. All data were ranked into quartiles. The analysis was performed using a Fine-Gray subdistribution hazard model. Adjusted subdistribution hazard ratios (adjusted SHR), corresponding 95% confidence intervals (CI), and respective p-values are presented for each follow-up period.

| **Characteristic** | **Timepoint** | **Adjusted SHR (95% CI)** | **P value** |
| --- | --- | --- | --- |
| Shannon's diversity | 12-month | 1.23 (0.93-1.62) | 0.14 |
|  | 90-day | 1.01 (0.70-1.48) | 0.94 |
| Pielou's evenness | 12-month | 1.07 (0.84-1.36) | 0.60 |
|  | 90-day | 0.97 (0.68-1.38) | 0.85 |
| Richness | 12-month | 1.07 (0.80-1.44) | 0.65 |
|  | 90-day | 1.02 (0.66-1.57) | 0.94 |
| Distance to centroid | 12-month | 1.02 (0.80-1.30) | 0.90 |
|  | 90-day | 1.01 (0.68-1.51) | 0.95 |
| Anaerobic species | 12-month | 1.13 (0.81-1.57) | 0.47 |
|  | 90-day | 1.11 (0.67-1.85) | 0.68 |
| Non-anaerobic species | 12-month | 0.97 (0.68-1.37) | 0.84 |
|  | 90-day | 0.94 (0.55-1.59) | 0.81 |

**Supplementary Table 3.** Cohort characteristics based on the detection of Enterobacterales in OP swabs, stratified by RTI diagnosis.

| **Variable** | **Enterobacterales** | **No Enterobacterales, RTI** | **No Enterobacterales, No RTI** |
| --- | --- | --- | --- |
|  | **n = 6** | **n = 48** | **n = 136** |
| Age, median [IQR] | 88.9 [84.7-92.6] | 88.7 [80.9-92.9] | 88.3 [81.8-92.6] |
| Female sex, n (%) | 2 (33.3) | 36 (75) | 97 (71.3) |
| Duration of residence, median (IQR) | 1254.5 [408-2476] | 538.5 [277.5-847.5] | 653 [242-1082] |
| **All-cause mortality, n (%)** | | | |
| 90-day | 0 (0) | 3 (6.3) | 8 (5.9) |
| 12-month | 0 (0) | 13 (27.1) | 22 (16.2) |
| **Comorbidities** | | | |
| Lung disease, n (%) | 4 (66.7) | 26 (54.2) | 39 (28.7) |
| Prior respiratory infection (12 months), n (%) | 4 (66.7) | 23 (47.9) | 33 (24.3) |
| Modified diet, n (%) | 0 (0) | 9 (18.8) | 33 (24.3) |
| Number of Rx-Risk comorbidities, median [IQR] | 6 [4-9] | 7 [5-8] | 4 [2-7] |
| **Medications dispensed within 90 days prior to sample collection, n (%)** | | | |
| Antibiotics | 3 (50) | 25 (52.1) | 43 (31.6) |
| Inhaled corticosteroids | 2 (33.3) | 8 (16.7) | 8 (5.9) |
| Proton pump inhibitors | 4 (66.7) | 28 (58.3) | 55 (40.4) |

**Supplementary Table 4.** Strains detected in contemporaneous gut and oropharyngeal (OP) samples collected from participants (01-01-002, 02-03-095, 03-04-006, 03-05-028) with Enterobacterales species detected in OP swabs. Reference strain names (from NCBI database) are presented.

| ***Escherichia coli*** | **Gut** | | **OP** |
| --- | --- | --- | --- |
| 01-01-002 | Nissle_1917_GCF_003546975.1 | | Nissle_1917_GCF_003546975.1 |
|  | KE48 | | N/A |
| 03-04-006 | SCU-124 | | SCU-124 |
|  | FDAARGOS_1291 | | N/A |
|  | Nissle_1917_GCF_003546975.1 | | N/A |
| 03-05-028 | M1_5 | | M1_5 |
| ***Klebsiella oxytoca*** | |  | |
| 02-03-095 | 86 | | 86 |
| ***Klebsiella pneumoniae*** | |  | |
| 03-05-028 | INF125 | | INF125 |
| ***Klebsiella variicola*** | |  | |
| 03-05-028 | FF907 | | FF907 |
